# Intuitive weights of harm for therapeutic decision making in smear-negative pulmonary Tuberculosis: an interview study of physicians in India, Pakistan and Bangladesh

**DOI:** 10.1186/1472-6947-14-67

**Published:** 2014-08-08

**Authors:** Chandrashekhar T Sreeramareddy, Mahbubur Rahman, HN Harsha Kumar, Mohsin Shah, Ahmed Manadir Hossain, Md Abu Sayem, Juan M Moreira, Jef Van den Ende

**Affiliations:** 1 Department of Population Medicine, Faculty of Medicine and Health Sciences, University Tunku Abdul Rahman, Sungai Long Campus, Bandar Sungai Long, Kajang 43000, Cheras, Selangor, Malaysia; 2 Institute of Epidemiology, Disease Control & Research (IEDCR), Dhaka, Bangladesh; 3 Department of Community Medicine, Kasturba Medical College (Manipal University), Mangalore, India; 4 Medical unit 2, Jinnah Hospital, Lahore, Pakistan; 5 Faridpur Medical College, Faridpur, Bangladesh; 6 National Tuberculosis Control Program (NTP), Rajshahi, Bangladesh; 7 Faculty of Medicine, Universidad Central del Ecuador, Quito, Ecuador; 8 Department of Clinical Sciences, Institute of Tropical Medicine, Antwerp, Belgium

**Keywords:** Tuberculosis, Treatment morbidity and mortality, Medical decision making, South Asia

## Abstract

**Background:**

To estimate the amount of regret and weights of harm by omission and commission during therapeutic decisions for smear-negative pulmonary Tuberculosis.

**Methods:**

An interviewer-administered survey was done among young physicians in India, Pakistan and Bangladesh with a previously used questionnaire. The physicians were asked to estimate probabilities of morbidity and mortality related with disease and treatment and intuitive weights of omission and commission for treatment of suspected pulmonary Tuberculosis. A comparison with weights based on literature data was made.

**Results:**

A total of 242 physicians completed the interview. Their mean age was 28 years, 158 (65.3%) were males. Median probability (%) of mortality and morbidity of disease was estimated at 65% (inter quartile range [IQR] 50-75) and 20% (IQR 8-30) respectively. Median probability of morbidity and mortality in case of occurrence of side effects was 15% (IQR 10-30) and 8% (IQR 5-20) respectively. Probability of absolute treatment mortality was 0.7% which was nearly eight times higher than 0.09% reported in the literature data. The omission vs. commission harm ratios based on intuitive weights, weights calculated with literature data, weights calculated with intuitive estimates of determinants adjusted without and with regret were 3.0 (1.4-5.0), 16 (11-26), 33 (11-98) and 48 (11-132) respectively. Thresholds based on pure regret and hybrid model (clinicians’ intuitive estimates and regret) were 25 (16.7-41.7), and 2(0.75-7.5) respectively but utility-based thresholds for clinicians’ estimates and literature data were 2.9 (1-8.3) and 5.9 (3.7-7.7) respectively.

**Conclusion:**

Intuitive weight of harm related to false-negatives was estimated higher than that to false-positives. The mortality related to treatment was eightfold overestimated. Adjusting expected utility thresholds for subjective regret had little effect.

## Background

Pulmonary Tuberculosis (PTB) is a major cause of morbidity and mortality worldwide, particularly in low-and-middle-income countries (LMICs) [1,2]. Early and optimal treatment of both smear-negative and smear-positive cases is the only effective strategy available for TB control [3]. Although an effective treatment is available, diagnostic methods such as sputum smear and sputum culture are not sensitive enough and facilities for culture are scarcely available in LMICs [3]. Physicians often face a dilemma when sputum smear and/or culture are negative in a suspected PTB patient, since several national guidelines recommend bacteriological confirmation before initiating the anti-TB treatment. However, the clinicians at referral (tertiary care) hospitals argue that most patients having clinical features of PTB but negative bacteriological tests deserve an adequate treatment [4]. Moreover, many referral hospitals are confronted with relatively more smear-negative PTB (SNPTB) cases than the lower level health facilities [5]. Clinicians are still reluctant to initiate treatment for SNPTB suspects due to non-availability of newer TB diagnostic tools [3,4]. Since the sensitivity of sputum culture is not higher than 60%, far too many false negative cases would be left untreated, if bacteriological proof were compulsory for treatment [4,6,7].

Pauker and Kassirer have recommended a decision threshold approach to solve conflicting issues such as treatment of SNPTB. They have defined the ‘decision threshold’ as a minimum required probability of the disease for allowing initiation of treatment. This approach is based on a compromise between factors associated with the treatment itself and those if the disease is left untreated [8]. ‘*Threshold*’ is defined as the (post-workup) probability of the disease at which the risk and cost of a diseased person left untreated equals the risk and cost of treatment of both the diseased and non-diseased. The probabilities and values of harm and benefit of a treatment must be taken into account to formally estimate the required level of certainty to justify a treatment [9]. This concept though has been applied to other medical conditions; so far only two studies have been published about SNPTB [5,10]. The first study of Basinga et al. from Rwanda has reported that physicians’ intuitive treatment threshold for SNPTB was 52.5% but a threshold based on literature data was only 2.8% [5].The second study, by Moreira et al. from four countries (Nepal, Ecuador, Laos and Rwanda) has reported that physicians’ intuitive weights (on a 10-point Likert scale) of harm by commission (treating a person who indeed is not diseased) and omission (not treating a person who is diseased) were 4.5 versus 10 respectively in therapeutic decision making for SNPTB [10].

Zikmund-Fisher et al. argue that the decisions related to life-and-death situations should consider emotional factors of both decision makers and patients who are affected by these decisions [11]. Hence these subjective factors not directly related to the disease and its treatment should be taken into account while making therapeutic decisions.

Generally patients’ perspectives are emphasised, but all important clinicians’ perspectives of therapeutic decisions are rarely studied. Previous studies on SNPTB (clinicians’ perspective) and parental perspectives for child vaccination against pertussis and influenza have highlighted that feelings of regret from provoked harm of medical interventions do affect the decision making [5,10,12,13]. A detailed analysis of the subjective (emotional) factors is useful in guiding clinicians in estimation of sound decision thresholds. Studies about weights of harm by omission and commission are important since correctness of therapeutic decisions has implications on patient care as well as on population health in LMICs and worldwide [10,14].

Regret in decision making can be investigated in different ways. Gross estimation of subjective regret for a commission or omission error can be given on a visual analog scale [15]. Expected harm of commission vs. omission can be calculated based on estimations of clinicians or literature data of mortality and morbidity related to disease and treatment via expected utility methodology [8]. Gross estimation (‘holistic approach’) might correspond to the ‘thinking fast’ (system I) and the expected utility method to the ‘thinking slow’ (system II) proposed by Kahneman [16]. Both are combined in the dual processing model proposed by Djulbegovic et al. [17]. In this model the authors add regret, which they limit to the system I, to the expected utility based analytical reasoning in an additive way. Regret can also be applied, not added to probabilities estimated by clinicians or found in the literature (hybrid model) [5,18]. Earlier research showed that the results of gross estimation might be very different from harm computed from clinicians’ estimations of mortality and morbidity, even after adjusting these estimations for omission/commission regret [5]. In this study, we apply the ‘hybrid model’.

India, Pakistan and Bangladesh are among the 22 high-burden TB countries, but so far no study has been done about physicians’ intuitive weights of harm during therapeutic decisions for SNPTB. We estimated the ‘weights of regret’ due to death resulting from omission or commission (as compared to natural death) in a suspected SNPTB patient. In parallel, to have an objective standard of comparison, we substituted literature probabilities for physicians’ intuitive estimates to calculate the weights of false positives and false negatives.

## Method

### Study design

A cross-sectional, interviewer-administered questionnaire survey.

### Setting and participants

Medical professionals from Bangladesh, India and Pakistan were selected for this survey. The participants had varying lengths of clinical experience and were working at different settings such as primary care, teaching hospitals attached to medical schools and tertiary care hospitals. The participants had not received any formal training in medical decision making. The main indicators for burden of TB and HIV, and indicators of TB control program for these countries are shown in Table 1[19].

**Table 1:** Characteristics of countries

**Characteristics**	**India**	**Pakistan**	**Bangladesh**
World Bank classification based on income	Lower middle	Lower middle	Low income
Total population (thousands)	1,241,492	176,745	150,494
TB mortality per 100,000 population-year (excludes HIV + TB)	24	33	45
TB prevalence 100,000 population-year (includes HIV + TB)	249	350	411
TB incidence 100,000 population-year (includes HIV + TB)	181	231	225
HIV prevalence in incident TB cases (%)	4.2	0.4	0.2
Incidence 100,000 population-year (HIV + TB)	7.6	0.84	0.42
Estimated% of new TB cases with MDR-TB	2.1	3.4	1.4
Estimated% of previously treated TB cases with MDR-TB	15	29	29
Percent new pulmonary cases smear- positive	65	50	82
Estimates of the case detection rate of new & relapse cases	59	64	45
Treatment success for all new cases (%) in 2010	89	90	91

Source: World Health Organization. Global tuberculosis report 2012.

### Sampling and sample size

From each country, we recruited medical professionals affiliated to a medical school who are likely to care for suspected TB patients in their clinical practice and physicians working at primary health care centers (in Bangladesh only). We did not use any formal sample size calculation.

### Questionnaire

The conceptual framework and study instrument we used was based on the study by Moreira et al. [10]. Only a few minor modifications were made. An English version of the questionnaire was administered to the participants during an interview conducted by the collaborating researchers from three countries. The questionnaire (see Additional file 1) contained information about demographics, years of experience, area of specialisation and case scenarios. The interviewers explained the case scenarios to each participant. The case scenarios explored the information about two main themes: i) perceived probabilities related to the disease and the treatment; and ii) intuitive values regarding the harm caused by the wrong decisions. In each of the case scenarios 1-5, the interviewers asked the participants to guess (perceived probabilities ranging from 0%-100%) about an estimated mortality and morbidity of untreated PTB and about the risk of morbidity and mortality caused by anti-TB treatment. In case scenario five, the participants were asked about the weights of regret arising from decisions namely; 1) forgoing treatment in a false negative case, 2) treating a false positive case, and 3) death related to a given decision despite this was based on optimal grounds. Weight of regret due to wrong decisions was estimated on a 10-point Likert scale while the weights of regret arising from death related to decisions was rated in comparison to natural death (death without any medical intervention) for which a weight of ‘1’ was assigned (refer to Additional file 1 for details). The cost of treatment was not estimated because anti-TB treatment is given free-of-cost for the patients under Directly Observed Treatment, Short course (DOTS) strategy in all three countries.

### Data collection

Between April and September, 2012 the research collaborators from each country interviewed the physicians. Ethical approval was obtained from research ethics committee of University Tunku Abdul Rahman, Selangor, Malaysia. Collaborators from India, Pakistan and Bangladesh, also obtained approval from ethics committees of the medical schools they were affiliated to. The potential participants were briefed about the research and informed consent was sought. Consenting physicians were interviewed face-to-face using the questionnaire. The researchers explained the scenarios to the participants and asked them to estimate the probabilities and weights of regret.

### Variables

Using the information about estimated probabilities and weights of harm some indicators were constructed according to methods used by Moreira et al. [10].

### Weight of a false negative (WFN)

$$\mathrm{WFN}=\left(\mathrm{dis}\_\mathrm{mort}+\mathrm{dis}\_\mathrm{morb}*w\_\mathrm{dis}\_\mathrm{morb}\right)\;*\;\mathrm{regr}\_\mathrm{pr}\_\mathrm{harm}\_\mathrm{om}$$

Where,

*dis_mort* = Mortality related with an untreated disease.

*dis_ morb* = Morbidity related with an untreated disease.

*w_dis _morb* = Weight of morbidity related with an untreated disease, which is the complement of the health status of a person affected by the disease compared with a healthy person of the same age (details about the question in the Additional file 1).

*regr_ pr_ harm_ om =* Regret for harm provoked by erroneous omission of the treatment.

### Weight of a false positive (WFP)

$$\mathrm{WFP}=\left(\mathrm{tr}\_\mathrm{mort}+\mathrm{tr}\_\mathrm{morb}*w\_\mathrm{tr}\_\mathrm{morb}\right)*\mathrm{regr}\_\mathrm{pr}+\mathrm{harm}\_\mathrm{tr}$$

Where,

*tr_mort* = Mortality due to a severe side effect of the treatment.

*tr_morb* = Morbidity due to severe side effects of the treatment.

*w_ tr _morb* = Weight of morbidity due to severe side effects of the treatment.

*regr_ pr_ harm_ tr* = Regret for harm provoked by the treatment (death in a non-diseased).

‘Calculated’ WFN and WFP were also computed without regret, and further by substituting estimated probabilities for literature data on mortality and morbidity (Table 2) into the equations given above. Further the ratios between weight of false negative (WFN) and weight of false positive (WFP) were calculated.

**Table 2:** Data from the literature on probabilities of mortality and morbidity related to the disease (TB) and treatment of tuberculosis

	**Median (%)**	**Range (%)**	**Number of studies retrieved**	**References**
Disease mortality	55	49-66	3	[20]
Disease morbidity	19	18-19	3	[21,22]
Treatment morbidity	5.8	1.8-12.5	12	[23-33]
Treatment mortality	0.09	0.02-0.4	6	[24,34-38]

The effect of data and estimations was incorporated finally in four thresholds: the first based on gross clinicians’ estimation of the weight of harm by commission vs. harm by omission; the second based on literature data [20-38]; a third based on estimation of probabilities of morbidity and mortality by the clinicians; and a last based on these estimations plus regret conditional on these probabilities, estimated by the clinicians.

### Data analysis

Data entry and analysis (see Additional file 2) was performed with SPSS (Statistical Package for Social Sciences) version 14.0 for windows. A comparison of estimated probabilities and weights was made between the countries and according to gender. The differences were tested for statistical significance using the Kruskal-Wallis test. A comparison between ratios of WFN/WFP calculated by four different approaches was made and the difference was tested for statistical significance using the Friedman test. Spearman’s rank correlation coefficient was used to test the relationship of ratios of WFN/WFP (both intuitive and calculated weights) with years of clinical experience. For all statistical tests a p-value of less than 0.05 was considered as significant.

## Results

A total of 242 clinicians were interviewed from three countries, namely, India (77), Pakistan (84) and Bangladesh (81). Overall median age of the participants was 28 years, female-to-male ratio 1/1.9. Median years of experience was 3 and 98 (40.5%) were specialist consultants (46 in internal medicine, the others in other specialties) (Table 3). Ten participants, six from India and four from Bangladesh were excluded from the analysis since they gave incomplete responses for either the probabilities or the weights of harm.

**Table 3:** Demographic characteristics of the respondents in India, Pakistan and Bangladesh

	**India (n = 77)**	**Pakistan (n = 84)**	**Bangladesh (n = 81)**	**All countries (n = 242)**
Age in years (median and IQR)	30 (26, 39)	24 (23, 26)	34 (29, 40)	28.0 (24, 36.3)
Experience in years (median and IQR)	3 (1.5, 8)	1 (0.5, 2)	9.0 (4.5, 14)	3.0 (0.8,9)
Sex (number and %)				
Male	46 (59.7)	47(56)	65 (80.2)	158 (65.3)
Female	31 (40.3)	37 (44)	16 (19.8)	84 (34.7)

### Estimated probabilities (%)

Overall median probabilities for mortality and morbidity from the disease were 65% (IQR 50-75) and 20 (IQR 8-30) and were significantly different among three countries. Overall median probability for occurrence of side effects, morbidity and mortality in case side effects occur was 10% (IQR 5-20), 15% (IQR 10-30) and 8% (IQR 5-20) respectively and were also significantly different among three countries (Table 4). Female participants estimated significantly higher probabilities for morbidity and mortality from the side effects (p = 0.009 and p = 0.031 respectively).

**Table 4:** Estimated probabilities (%) of morbidity and mortality from untreated SNPTB and side effects in treated SNPTB (median and inter quartile range)

		**India (n = 77)**	**Pakistan (n = 84)**	**Bangladesh (n = 81)**	**All countries (n = 242)**
1	Mortality from untreated SNPTB*	70 (65-75)	60 (30-82.5)	60 (40-70)	65 (50-75)
2	Morbidity from untreated SNPTB¶	5 (5-10)	20 (10-30)	29.5 (20-30)	20 (8-30)
3	Probability of side effects occurring if SNPTB is treated¶	6 (3-9)	10 (5-20)	15 (10-29)	10 (5-20)
4	Conditional probability of death due to side effects if they occur when SNPTB is treated¶	5 (3-5)	10 (4.5-20)	12.5 (5-25)	8 (5-20)
5	Conditional probability of morbidity as a result of side effects when SNPTB is treated¶	10 (7-15)	25 (10-50)	15 (10-30)	15 (10-30)
6	Absolute probability of mortality arising from side effects of anti-TB treatment¶×	0.25 (0.11-0.45)	1.5 (0.71-10)	1(0.22-4.0)	0.70 (0.23-3.1)
7	Absolute probability of morbidity arising from side effect of anti-TB treatment¶π	0.6 (0.3-1)	3.0 (1-7.4)	3 (0.57-8)	1.5 (0.6-4.6)

¶p < 0.001.

× Calculated as row 3 × row 4.

π calculated as row 3 × row 5.

*p < 0.01.

### Estimated weights (relative to perfect health) of morbidity related with disease and treatment

Overall median estimated weights (%) of morbidity related to disease and treatment (as a complement of health status of a person who is diseased as compared to a healthy person; case scenarios 4 & 5 in the questionnaire) were 25% (IQR 15-40) and 60% (IQR 40-80) respectively and the weights were significantly different among three countries. The median weights estimated for regrets of death due to omission, provoked but unjustified death and provoked but justified death were 4 (IQR 3-5), 3 (IQR 2-4) and 1 (IQR 1-2) respectively and the difference between three types of estimated weights was statistically significant. The differences in three types of regret between the countries were also statistically significant (Table 5).

**Table 5:** Estimated weights of mortality and morbidity and values of regret in all the countries

	**India (n = 77)**	**Pakistan (n = 84)**	**Bangladesh (n = 81)**	**All countries (n = 242)**
Weight of morbidity related to the disease (%)¶	20 (10-30)	30 (17.5-50)	35 (20-45)	25 (15-40)
Weight of morbidity related to treatment (%)¶	80 (75-90)	50 (20-60)	50 (40-80)	60 (40-80)
Regret due to provoked but justified death (relative to natural death)¶	1(1-2)	1 (1-2)	2 (1-2)	1 (1-2)
Regret due to provoked but unjustified death (relative to natural death)*	3 (2.5-4)	2 (1-3)	3 (2-5)	3 (2-4)
Regret of death due to omission (relative to natural death)¶	4 (4-5)	3 (1-3.5)	4 (3-7)	4 (3-5)

*p < 0.05, ¶ p < 0.001.

### Intuitive weights of false positive and false negatives

Overall medians for intuitive WFN and WFP were 9 (IQR 7-10), and 3 (IQR 1-6) respectively. The median intuitive WFP was significantly different among three countries whereas median intuitive WFN was similar in three countries. In Bangladesh, seven participants had estimated intuitive WFP as zero and six participants had estimated intuitive WFN as zero.

Overall median calculated WFN and WFP based on literature data, without regret were 59 (57-62) and 3.5 (2.4-4.7). Overall median calculated WFN and WFP based on intuitive estimates without regret were 71 (57-81) and 1.7 (0.6-5.8). Overall median calculated WFN and WFP based on intuitive estimates adjusted for regret were 252 (148-336) and 5 (1.83-17.29) (Table 6).

**Table 6:** Weights of false negatives and false positives

	**India (n = 77)**	**Pakistan (n = 84)**	**Bangladesh (n = 81)**	**All countries (n = 242)**
Intuitive weight of false negative^€^	9 (8-10)	9 (5-10)	8 (7-10)	9 (7-10)
Intuitive weight of false positive	1 (1-2)	5 (2-7)	4.5 (2-6)	3 (1-6)
Calculated weight of false negatives based on literature data (without regret)	58 (56-60)	60 (58-64)	61 (58-63)	59 (57-62)
Calculated weight of false positives based on literature data (without regret)	4.7 (4.4-5.3)	2.9 (1.2-3.5)	2.9 (2.4-4.7)	3.5 (2.4-4.7)
Calculated weight of false negatives based on intuitive estimates without regret	71 (65-76)	74 (48-90)	68 (56-79)	71 (57-81)
Calculated weight of false positives based on intuitive estimates without regret	0.7 (0.4-1.2)	3.3 (.9-7.8)	3.9 (1.5-14)	1.7 (0.6-5.8)
Calculated weight of false negatives (WFN) (based on intuitive estimations), regret included	302 (241.87-347.75)	291 (189.0-367.8)	129 (83.25-271.05)	252 (148-336)
Calculated weight of false positives (WFP) (based on intuitive estimations), regret included	2.02 (1.06-4.14)	15.64 (5.1-50.27)	5.6 (1.81-16.93)	5 (1.83-17.29)

^€^p > 0.05.

### WFN/WFP Ratios and thresholds

**Figure 1:**
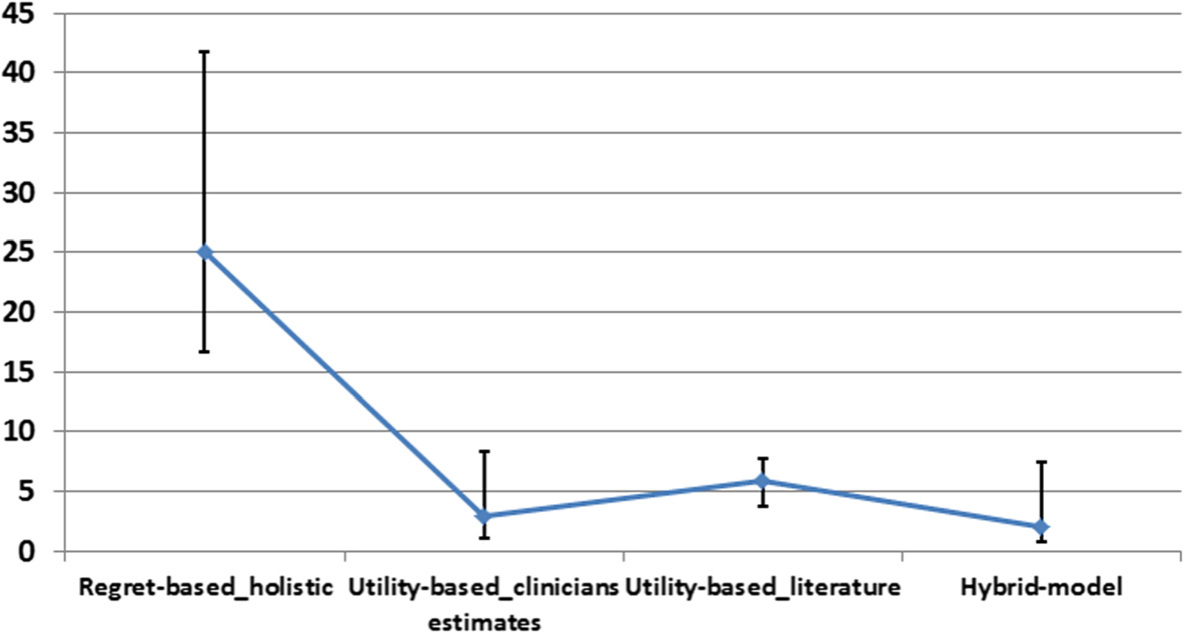
**Thresholds (median and quartiles) for treatment of smear-negative PTB calculated by four different approaches.** Regret-based holistic: threshold calculation on the basis of regret estimations of omission and commission on a Likert scale (pure regret based). Utility-based clinicians’ estimates: threshold based on clinicians’ intuitive estimates for disease and treatment morbidity and mortality without clinicians’ regret. Utility-based literature: threshold based on literature data for disease and treatment morbidity and mortality without clinicians’ regret. Hybrid model: threshold based on clinicians’ intuitive estimates for disease and treatment morbidity and mortality combined with clinicians’ regret for death by omission or commission.

The omission vs. commission harm ratio based on intuitive weights given on a 10-point Likert scale was 3.0 (1.4-5.0), corresponding to a threshold of 25%. This ratio based on expected utility theory with weights calculated with literature data was 16 (11-26), with a threshold of 5.9. The same with estimations by the clinicians instead of literature data gave a ratio of 33 (11-98) and a threshold of 2.9%. The hybrid approach, where regret for an unjustified death was applied to mortality estimations by the clinicians gave a ratio of 48 (11-132), with a threshold of 2 (Table 7 and Figure 1).

**Table 7:** Ratios between weights of false negatives versus weights of false positives by four different methods¶

	**India (n = 77)**	**Pakistan (n = 84)**	**Bangladesh (n = 81)**	**All countries (n = 242)**	**Threshold all countries**
Intuitive WFN/Intuitive WFP	8 (4.5-9)	2 (1.4-3.3)	1.4 (0.97-2.54)	3 (1.4-5)	25 (16.7-41.7)
Calculated WFN/calculated WFP based on literature data without regret	12 (11-13)	19 (13-26)	22 (18-52)	16 (12-26)	5.9 (3.7-7.7)_
Calculated WFN/calculated WFP based on intuitive estimates without regret	105 (54-141)	17 (4.6-34)	21 (8-56)	33 (11-98)	2.9 (1-8.3)
Calculated WFN/calculated WFP based on intuitive estimates with regret	131.62 (72.89-282.08)	19.04 (6.05-48.97)	28.42 (7.84-76.88)	48.69 (12.28-132.0)	2.0 (0.75-7.5)

¶Difference between ratios: p < 0.001.

Years of experience were negatively correlated with both clinicians’ estimated and calculated WFN and WFP. Spearman’s correlation coefficients ranged from -0.046 to -0.229 (details not shown).

## Discussion

### Results

The participants in three countries estimated the probabilities of morbidity and mortality from disease quite accurately since the estimated probabilities were comparable to the literature data. The mortality of treatment was over estimated by eight times. The intuitive estimated weight of regret about omission (false negative) was three times higher than the weight of regret about commission (false positive). This is in line with earlier research [14]. Even when WFN and WFP were calculated by other methods i.e. based on intuitive estimates of morbidity, mortality and literature probabilities, the WFN was much higher than the WFP in three countries. Most estimates of the probabilities, WFN and WFP were significantly different among the countries. The probabilities and weights of harm were not significantly different between male and female participants. There was a negative relationship (correlation) between WFN and WFP and years of clinical experience.

In general, the young physicians in our study considered that it is less harmful to commit than to omit which is similar to the results of the study by Moreira et al. from four LMICs [10]. Previous research has also shown that medical decisions generally are favorable towards treatment and the decisions depend on the perspective of the decision maker as found in our study [11]. Young physicians in our survey may have given a higher weight to harm by omission considering the potential benefit of the treatment available for PTB under DOTS strategy adopted in most LMICs. In Moreira’s study [10], physicians from Laos had given a consistently high value of 10 for WFP, but physicians in our study estimated a lower WFP. Moreira et al. suggest that strict national guidelines about smear positivity for initiation of anti-TB treatment as the reason for high WFP estimated by the Laotian physicians. Currently, the national guidelines in most LMICs do not strictly require smear-positivity to start anti-TB treatment; instead the guidelines provide a separate category and treatment regimen for SNPTB.

Despite fairly accurate estimation of baseline probabilities for disease morbidity and mortality by the physicians, they are less likely to integrate them into the decision threshold because the estimated baseline probabilities for treatment mortality were eightfold higher than reported in literature data. A similar conclusion was made by Moreira et al. since the physicians in their study also overestimated the treatment mortality by tenfold. This was also supported by their previous study from Rwanda [5]. In the Rwandan study the influencing factors for treatment threshold estimated by the physicians were close to the literature data but they had wrongly integrated their estimations into a final threshold for SNPTB [5].

### Limitations

Our survey had some noteworthy limitations which should be considered while interpreting the results. We selected a convenient sample of physicians who had limited experience of caring for TB patients. The sample was not homogenous in terms of demographics since a higher proportion of physicians were males, particularly in Bangladesh and the participants were relatively young. Despite this, most probabilities and weights did not significantly differ by gender. We did the survey among physicians in only one location in India and Pakistan. The participants may not be representative of all the physicians likely to provide healthcare to TB patients in each country. Hence the results cannot be generalized to the entire country or to countries other than those in which the survey was done.

The estimated probabilities and weights were for PTB, which is a disease with a lower threshold. We used PTB as an example to understand the role of weights and probabilities while making medical decisions. If we had used an example of a medical condition whose treatment was less effective and had more serious side effects, the physicians might have shown a higher tendency to omit treatment. Hence our findings cannot be generalised to all the disease conditions with varying mortality and morbidity related to disease and treatment.

The weight of morbidity from treatment was overestimated relative to the weight of the disease possibly due to non-comprehension of the case scenario or to the inappropriate integration of regret in their responses.

The estimation of the regret of omission and commission was done on a scale from 1-10, with the permission to go over the limit of 10. Other authors prefer a scale from 1-100, with clear limits. A 1-10 scale is bound to generate a ceiling effect [15].

### Impact

Omission and commission biases and emotionally-driven regret should be emphasized during decision making. Studies about emotional factors of physicians i.e. weights of regrets of harm due to omission or commission and the role of estimated probabilities in medical decision making are lacking from LMICs. Studying these factors in LMICs where no formal training in medical decision making exists may be useful in training of young physicians. Although appropriateness of formal inclusion of emotionally driven factors such as regret into decision making is discussed, physicians should be informed about their importance. Awareness of the emotionally driven factors in medical decision could improve the quality of decisions and minimize the errors by omission or commission.

### Future research

Future studies should estimate the baseline probabilities, weights of regrets and treatment thresholds for other conditions of varying morbidity and mortality from disease and treatment. Other ways of estimating thresholds, like the regret-based or the dual- processing could be addressed also [15,17,39]. Finally, further studies should assess the impact of training in medical decision making on quality of medical decisions.

## Conclusion

Intuitive weight of harm related to false-negatives was estimated higher than that of false-positives. However the mortality related to treatment was eightfold overestimated. Although the observed effect of adjusting WFN and WFP for clinician’s regret, computed in accordance to expected utility theory, was low in this study, awareness of the emotional factors should be included into clinical decision training.

## Competing interests

The authors declare that they have no competing interests.

## Authors’ contributions

CTS: Conceived the research, performed the data analyses, and wrote the first draft of the manuscript. MR: interviewed the participants, analysed the data, co-drafted the manuscript. HNHK: interviewed the participants, analysed the data, co-drafted the manuscript. MS: interviewed the participants, analysed the data, co-drafted the manuscript. AMH: interviewed the participants, analysed the data, co-drafted the manuscript. MAS: interviewed the participants, analysed the data, co-drafted the manuscript. JMM: prepared the questionnaire, assisted in writing the methods section and commented on the earlier drafts of the manuscripts. JVE: prepared the questionnaire, assisted in writing the methods section and commented on the earlier drafts of the manuscripts. All authors read and approved the final manuscript to be submitted for publication.

## Pre-publication history

The pre-publication history for this paper can be accessed here:

http://www.biomedcentral.com/1472-6947/14/67/prepub

## Supplementary Material

Additional file 1 Questionnaire used for interviewing the physicians. Click here for file

Additional file 2 Original database for demographic factors and responses provided by physicians. Click here for file
