# Supplementary material for: Intuitive weights of harm for therapeutic decision making in smear-negative pulmonary Tuberculosis: an interview study of physicians in India, Pakistan and Bangladesh
Source: BMC Med Inform Decis Mak. 2014 Aug 8;14:67. doi: 10.1186/1472-6947-14-67 (PMC4127046; doi:10.1186/1472-6947-14-67)
Supplement: Additional file 1 — Questionnaire used for interviewing the physicians. [file 1472-6947-14-67-S1.docx]

# Additional file 1

**QUESTIONNAIRE**

Dear colleague:

This questionnaire was prepared for a study aimed at understanding major concepts in medical decision making in smear negative tuberculosis. This questionnaire has also been applied in some other countries of Asia, Central Africa and South America.

The obtained results will serve to adapt the training methods in medical decision making. Please follow carefully the instructions and be clear if you make amendments. Thank you in advance for your kind cooperation.

| Age :...... years |  | Gender(M or F) : |  |
| --- | --- | --- | --- |
| Years of experience since your graduation as doctor:................................... | | | |
| Specialization (if any):.................................. | | | |

Case scenario-1

Suppose a patient with pulmonary tuberculosis remains untreated, as if it was the time when specific chemotherapy was not yet discovered. In such scenario one of the following outcomes can occur to the patient: 1) die of tuberculosis, 2) survive with symptoms related with the disease, or 3) spontaneous remission of symptoms (absence of symptoms).

According to your experience and knowledge, please guess the probability (frequency) for each of above outcomes in such a case scenario. Note that the sum must be 100.

| Outcome | Probability |
| --- | --- |
| Death | % |
| Survival with lifelong symptoms | % |
| Spontaneous remission | % |
| Total | 100 % |

Case scenario-2

Currently a specific treatment is available. In most of the countries a standard schema of 2 months with daily rifampicin, isoniazid, pyrazinamide and ethambutol followed by 4-6 months of rifampicin and isoniazid is recommended. A patient receiving such a treatment can develop severe side effects, like clinical hepatitis. What is the probability of any such an outcome? Please don’t give intervals (ranges), please try to guess a number.

| Standard treatment | Probability of severe side effects |
| --- | --- |
| RHZE for 2 months, then RH for 4 months | % |

Case scenario-3

If severe side effects occur the patient can die, but the patient can also survive with or without lifelong symptoms related to treatment with e.g., cirrhosis. Please guess the probability of each of these outcomes to occur. Please don’t give intervals (ranges), please try to guess a number. Note that the sum must be 100.

| Outcome | Probability |
| --- | --- |
| Death | % |
| Survival with lifelong symptoms | % |
| Remission without symptoms | % |
| Total | 100 % |

Case scenario-4

A diseased person surviving with lifelong symptoms because he was not treated has an impaired health status, compared with the expected health status for a healthy person at the same age. For instance, one could say that a tetraplegic person has only 10% of the expected health status compared with a healthy person at the same age. Similarly a hemiplegic patient could have 25% of his expected health status.

Please guess the percentage of the expected health status of a person surviving with symptoms of untreated tuberculosis. These symptoms may include ongoing fever, shortness of breath sometimes needing supplementary oxygen to comply common tasks, depression, rejection by relatives, etc.

| Percentage of the expected health status for somebody surviving with symptoms because he was not treated for tuberculosis. | % |
| --- | --- |

Case scenario-5

The health status of somebody surviving after drug related severe side effects is also less than the expected, compared with a healthy person of the same age. Please guess what is the percentage of the expected health status for somebody with such a condition. This health condition due to severe side effects of treatment may include cognitive and mobility impairment related with cirrhosis.

| Percentage of the expected health status for somebody surviving after drug related severe side effects. | % |
| --- | --- |

Case scenario-6

Now suppose you are in charge of a patient. You obtained all pertinent and available clinical information from the patient, which included results of three sputum smear examinations for acid-fast bacilli (AFB) which were negative. However you still suspect that the patient has pulmonary tuberculosis, but you are uncertain about the true diagnosis. In such a situation among other possibilities, you can err in two ways:

1) treating someone not having tuberculosis, or 2) abstaining from treatment for somebody who is actually having tuberculosis. We assume that the cost of drugs is free of cost, under TB control program and it is not taken into account.

Rate from 0 to 10 the “regret” you would have doing the mistake of giving an antituberculous treatment to somebody who does not actually have pulmonary tuberculosis. (Don’t hesitate to surpass 10 if you consider that it is necessary)

| No regret | | | | | | Large regret | | | | |
| --- | --- | --- | --- | --- | --- | --- | --- | --- | --- | --- |
|  |  |  |  |  |  |  |  |  |  |  |
| 0 | 1 | 2 | 3 | 4 | 5 | 6 | 7 | 8 | 9 | 10 |

Now rate from 0 to 10 the “regret” you would have by making the mistake of abstaining from treatment for somebody who is actually having pulmonary tuberculosis. (Don’t hesitate to surpass 10 if you consider necessary)

| No regret | | | | | | Large regret | | | | |
| --- | --- | --- | --- | --- | --- | --- | --- | --- | --- | --- |
|  |  |  |  |  |  |  |  |  |  |  |
| 0 | 1 | 2 | 3 | 4 | 5 | 6 | 7 | 8 | 9 | 10 |

After taking your decision there is a chance that your patient dies, despite the fact that your decision was based on optimal grounds. Such an outcome could lead to the following reflection.

What is the “weight of regret” we have for being involved in such death? Let’s say that a natural death (somebody dying without a specific medical intervention) has a weight equal to “1”.What would be the weight of a death resulting of a side effect of a treatment that was prescribed by you? E.g.: if your answer is “2” it means that you consider a provoked death being 2 times worse than a natural one.

Now give your own rates for the following situations:

| 1. Weight of a provoked but justified death (a death resulting of a side effect of treatment in somebody who had tuberculosis) |  |
| --- | --- |
| 1. Weight of a provoked but unjustified death (a death resulting of a side effect of treatment in somebody who didn’t need the treatment because he/she didn’t have tuberculosis) |  |
| 1. Weight of a death provoked by an erroneous omission of treatment in somebody who had tuberculosis (death as a result of complications of tuberculosis). |  |

Thank you very much
